# Fern-extract in Tapeworm

**Published:** 1896-04

**Authors:** 


Fern-extract as a Remedy for Tapeworm. An interesting case of poisoning has just happened in Augsburg. A physician prescribed fern-extract for a servant girl suffering with tapeworm, and gave her a relatively very small dose: 8 g. Very doubtful symptoms followed very soon. The girl was seized with cramps and died with epileptic symptoms which pointed clearly to poisoning. The physician reported the case to the authorities at once. The body was subjected to a post-mortem examination, with what result is not yet known. Similar effects have happened before with the same medicine, but only rarely. The matter is of especial importance, inasmuch as fern-extract is a very common remedy for tapeworm.—*Thierärzt Centralblatt*, December 15, 1895.

